# Ergonomic assessment of postal workers’ pain symptoms and musculoskeletal risks related to parcel processing activity for delivery

**DOI:** 10.17179/excli2022-4857

**Published:** 2022-04-27

**Authors:** Lincoln Silva, Nélson Costa, Carolina Schutz Rosa, Irandir Izaquiel Paulo, Natália Mattos da Silva, Cesar Giracca, Sabrina da Silveira Simões, Adriana do Nascimento Aquini, Giselle Merino, Eugenio Andrés Díaz Merino

**Affiliations:** 1 ALGORITMI Research Centre (Portugal). Minho University - Azurém, Guimarães, PT; 2 Universidade Federal de Santa Catarina, Florianópolis, SC; 3 Empresa Brasileira de Correios und Telégrafos (ECT), Brasilia

**Keywords:** musculoskeletal disorders, parcel processing, motion capture, anthropometric data

## Abstract

The parcel delivery activity is carried out all over the world and workers in this sector have suffered from musculoskeletal disorders (MSDs) due to the strong demand for work generated by the recent increase in e-commerce. This study aimed to evaluate postal workers' pain symptoms, movements and identify MSDs risks related to the parcel processing activity for delivery, proposing preventive measures. A sample of thirty-two workers was evaluated with the application of sociodemographic and Nordic questionnaires and electrical bioimpedance. The motion capture sensors were used to evaluate right/left shoulder joints, segment C7-T1 (Cervical) and segment L5-S1 (Lumbar) of three postal workers (percentiles of anthropometric data: 5, 50, and 95) during four real work activities that are part of the parcel processing. The analyzed workers presented musculoskeletal complaints in practically all body regions, with a greater prevalence in shoulders, hands, lower back, and knees. According to the Body Mass Index (BMI), they were on average overweight (27.8 ± 3.7 kg/m^2^). In the movement analysis, we identified risks related to cervical protrusion, anterior trunk flexion, and shoulder flexion, in addition to repetitive movements. In some activities, the higher stature showed an increase in lumbar and cervical anterior flexion. The set of evaluations showed that the activity of processing orders for delivery offers musculoskeletal risks. We identify that ergonomic adaptations are necessary to adapt the heights of the work environment to the statures of the postal workers. Relevance to industry: The activity of processing orders for delivery is carried out practically all over the world generating jobs and income for its employees. Nonetheless, there are still situations of ergonomic disadvantage that can generate musculoskeletal risks. The findings elucidate ergonomic risks and provide useful information for future ergonomic interventions in the postal/delivery workplace environment.

## Introduction

Postal service is one of the oldest communication services of humanity (BPMA, 2006[4]), and in Brazil corresponds to the country's historical transformation as the activities that previously involved only letter delivery today involve complex parcel delivery services (ECT, 2019[8]). Parcel delivery service has increased its activities in response to the growth of the purchase from e-commerce. The Brazilian postal service company (ECT) revealed in annual financial reports revenue growth (3.27 %) from 2017 to 2018, mainly driven by the parcel segment that corresponds to approximately 60 % of all current delivery activities (ECT, 2019[10]). In 2020, during the COVID-19 pandemic, Brazil documented a 41 % increase in invoicing of e-commerce operations and 30 % in the volume of online orders. The country currently has almost 80 million digital consumers (ECT, 2021[7]).

Amid the slowing economic activity, the pandemic of the new coronavirus has accelerated the growth of e-commerce worldwide and increased demand for parcel deliveries, especially in developed and developing countries (UNCTAD, 2021[34]). Also, according to the United Nations Conference on Trade and Development, online shoppers prefer home delivery over store collection.

Domestic e-commerce deliveries rely on national postal services, and the postal service workers are the ones that handle home delivery for a considerable number of customers. These workers are commonly affected by musculoskeletal disorders (MSDs) due to cargo handling, repetitive movements, vibration, and awkward postures during the workday (Harcombe et al., 2009[14]; Mattioli et al., 2011[22]; Hurley et al., 2012[15]). In Brazil, ECT workers are among the most affected by work-related illnesses and accidents (Filgueiras, 2017[11]). ECT has published an official management report for 2017, citing that among 110,000 workers, about 8.4 % are in absenteeism situations (ECT, 2018[9]).

Although MSDs in postal workers are a reality in many countries (Harcombe et al., 2009[14]; Hurley et al., 2012[15]; Swedish Work Environment Authority, 2016[32]; Tiwari and Agarwal, 2018[33]), there is a lack of studies that focus on the carried-out activities before the workers leave for the delivery route. These activities are related to sorting by destination, organizing orders, and placing them into the delivery vehicle.

Parcel delivery companies value agility in the delivery and preservation of order integrity. However, there is a human cost for the service to meet those standards. Although parcel delivery activities have economic representativeness, the impact of these activities on workers' health requires a better understanding of symptoms and which activities can lead to MSDs and absenteeism. Therefore, this study aimed to evaluate postal workers' pain symptoms, their task-related movements and identify MSDs risks related to the parcel processing activity for delivery, proposing preventive measures at a Postal Delivery Center located in southern Brazil. The research included three stages: problem investigation, data collection, and data analysis. In this way, data collection procedure was composed of five steps: (1) consent signatures and surveys; (2) definition of the activities that would be evaluated, based on the data obtained from the movement analysis, workers' complaints, Nordic Questionnaire, and direct observation; (3) bioimpedance analysis; (4) motion capture (Xsens); and (5) activity data recording.

## Materials and Methods

### Participants

In the evaluated parcel processing unit, there was a predominance of male sex workers. In this way, the sample consisted of 32 male workers who met the study inclusion criteria (worked for more than 12 months performing the order processing for the delivery activity for more than 4 hours a day). All assessed workers answered the questionnaires and underwent electrical bioimpedance. From this group, three subjects were chosen, based on their anthropometric data, to represent the anthropometric profiles in the study of the movements. This is a continuation of a previous paper on Ergonomic Assessment of Musculoskeletal Risks in Brazilian postal workers (Silva et al., 2020[29]) and intends to get a wider view of musculoskeletal disorders regarding the activity of parcel processing for delivery.

### Technical procedures

Technical procedures involved the application of sociodemographic and Nordic Questionnaire, bioimpedance evaluations, and motion capture. Motion capture occurred during the parcel processing activity for delivery in real-time (Merino et al., 2019[23]) with three postal workers selected to represent different anthropometric percentiles (5, 50, and 95). Two of them are right-handed (percentiles 5 and 95) and one of them is left-handed (percentile 50).

The heights of the postal workers were 157 cm (percentile 5), 176 cm (percentile 50), and 181 cm (percentile 95). According to Iida and Guimarães (2016[16]), the data from static anthropometry is recommended for dimensioning products and workplaces that involve small body movements.

However, real-work activities are dynamic and involve larger movements making adjustments necessary to accommodate most body movements.

All 32 workers undergo regular health assessments so that they can develop their activities in good health conditions. At the time of the survey, none of the selected workers have any abnormal health conditions that prevent them from performing their work.

**Figure 1:**
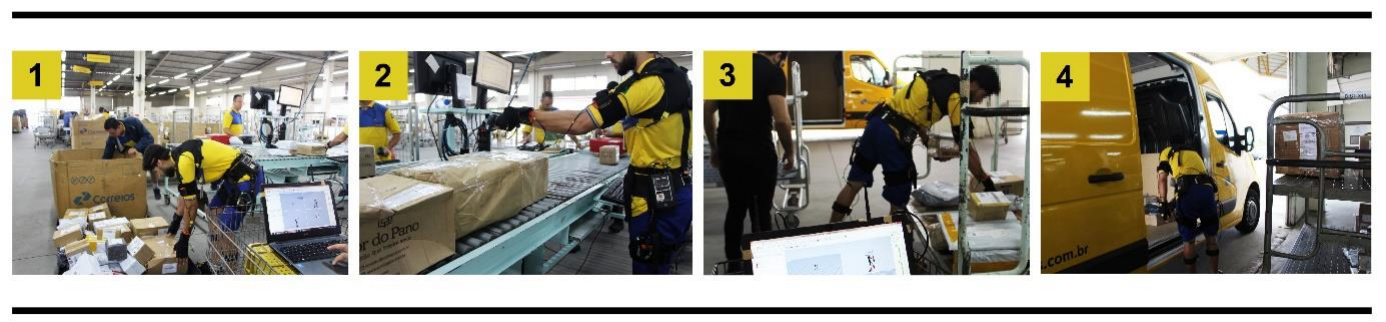
Work activity performed on four different workstations

The activities performed are part of the parcel processing process and were divided into (1) Container parcel removal, (2) Barcode reading, (3) Cargo destination sorting, and (4) Vehicle loading. Figure 1(Fig. 1) shows respectively the four work activities observed.

#### Sociodemographic questionnaire

The sociodemographic questionnaire was used to define the social characteristics of the sample. Information was collected on age, body mass, height, service time in parcel delivery (time counted from the admission in the company to the assessment date), and dominant member.

#### Nordic questionnaire for musculoskeletal symptoms

The nordic questionnaire for musculoskeletal symptoms (Kuorinka et al., 1987[17]) is a reliable instrument that has been translated and validated for the Brazilian population (Pinheiro et al., 2002[26]).

#### Electrical bioimpedance (BIA)

For this purpose, the instrument used was a portable bioelectrical impedance scale OMRON® HBF-514C. This equipment has four electrodes bilaterally. The current used is 500 µA at a frequency of 50 kHz. This instrument was used to determine the body fat and muscle percentage.

#### Motion capture

Motion capture by inertial sensors was used to analyze joint amplitudes and execution time in four different workstations. The collection was performed with equipment composed of 17 inertial sensors attached to different parts of the body (Xsens MVN Biomech TM, Enschede, the Netherlands) that track the segments, orientation, position, movement, and center of mass. Body structures analyzed were: i) Right/Left shoulder joints, ii) segment C7-T1 (Cervical), and iii) segment L5-S1 (Lumbar). The system operates in real-time and captures samples at a frequency of 120 Hz. Each activity was assessed at periods of 30 seconds.

### Statistical analysis

Sociodemographic, occupational, and clinical characteristics were described using mean and standard deviation (parametric data), median, quartile deviation, absolute frequencies, and percentages (non-parametric data). 

The relationship or strength of association between the variables was determined using correlation coefficient. As the variables had normal distribution, Pearson's correlation coefficient was used. Thus, tests of correlations between age, body mass, height, BMI, percentage of body fat, percentage of muscle, service time in delivery of orders were carried out.

All statistical analyzes were performed using the Statistical Package for Social Sciences (SPSS v. 272.0) program, considering a significance level of 5 %.

## Results

### Sociodemographic questionnaire 

Concerning the sociodemographic questionnaire, the 32 workers participating in the sample were all male, with an average age between 43.7 ± 7.34 (Min.: 27; Max.: 53) years, an average service time of 18.8 ± 8.4 (Min.: 5; Max.: 33) years, an average body mass of 85.4 ± 13.6 kg (Min.: 67.1; Max.: 123.0), and an average height of 174.9 ± 6.9 cm (Min.: 156; Max.: 190).

The participants were 93.8 % right-handed and 6.3 % left-handed. Service providers within a period ranging between 5 and 33 years. Anthropometric and BIA data are shown in Table 1(Tab. 1) considering their specifications.

A positive correlation was found between service time and BMI (p= 0,04; r= 0,36) and the percentage of body fat (p= 0,04; r= 0,38) while there was a negative correlation between service time and the percentage of muscle (p=0,006; r=-0,48). Thus, in the studied group, service time may have influenced more weight gain and the loss of muscle mass than expected, since parcel delivery is mainly a physical demanding task.

### Nordic questionnaire for musculoskeletal symptoms

The Nordic Questionnaire was applied to assess the musculoskeletal symptoms of the 32 postal workers. All responded to the questionnaire, representing a 100 % participation rate. About the annual and weekly presence of musculoskeletal pain symptoms, it was found that out of 32 workers, 100 % reported pain symptoms in at least one region of the body. There were no reports of absence of symptoms in the periods questioned.

Regarding the occurrence of pain, the most mentioned body parts were shoulders (69 %), lumbar (59 %), wrist/hands (56 %), knees (44 %), feet/ankles (41 %), neck (31 %), dorsal (28 %), hip/thigh (28 %) and elbows (25 %), in the last 12 months. When asked if they were prevented from performing normal activities such as work, domestic and leisure activities in the last 12 months, the regions most mentioned for functional disability were lumbar (41 %), followed by shoulders (31 %), knees (28 %), hip/thigh (25 %), back (22 %), wrist/hand (22 %), feet/ankles (22 %), neck (13 %) and elbows (6 %).

**Figure 2:**
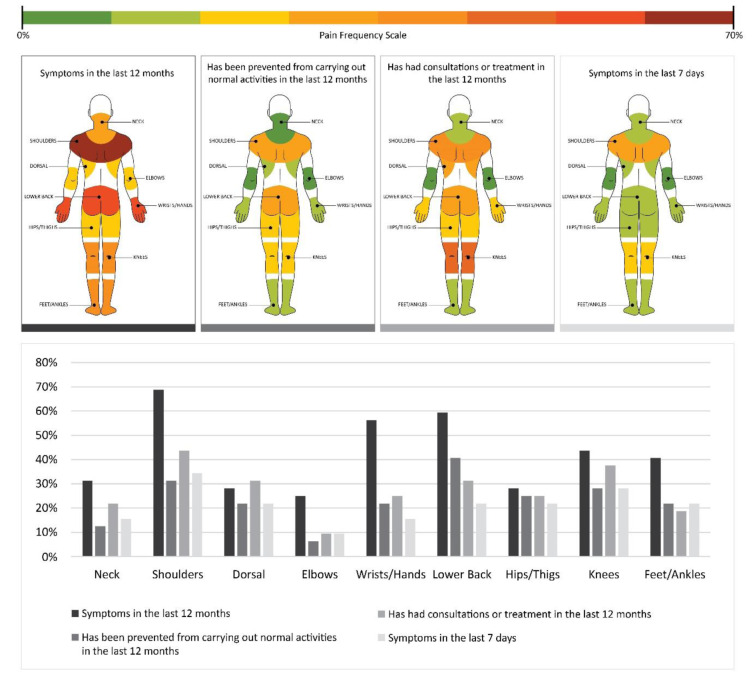
Anatomical region and percentage of pain frequency according to the Nordic Questionnaire

When asked about health professionals' appointments and treatments in the last 12 months, the most cited body regions were: shoulders (44 %), knees (38 %), lumbar (31 %), back (31 %), wrist/hand (25 %), hips (25 %), neck (22 %), feet/ankles (19 %) and elbows (9 %). Respecting presence of symptoms (pain, tingling/numbness) in the last seven days, the most reported regions were shoulders (34 %), knees (28 %), dorsal (22 %), lumbar (22 %), hip/thigh (22 %), feet/ankles (22 %), neck (16 %), wrist/hand (16 %) and elbows (9 %) (Figure 2(Fig. 2)).

### Motion capture

#### Right/Left shoulder joints

**Figure 3:**
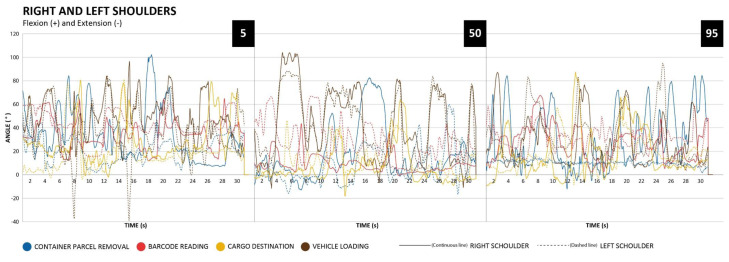
Right and left shoulders flexion/extension movements, percentile 5, 50 and 95

**Figure 4:**
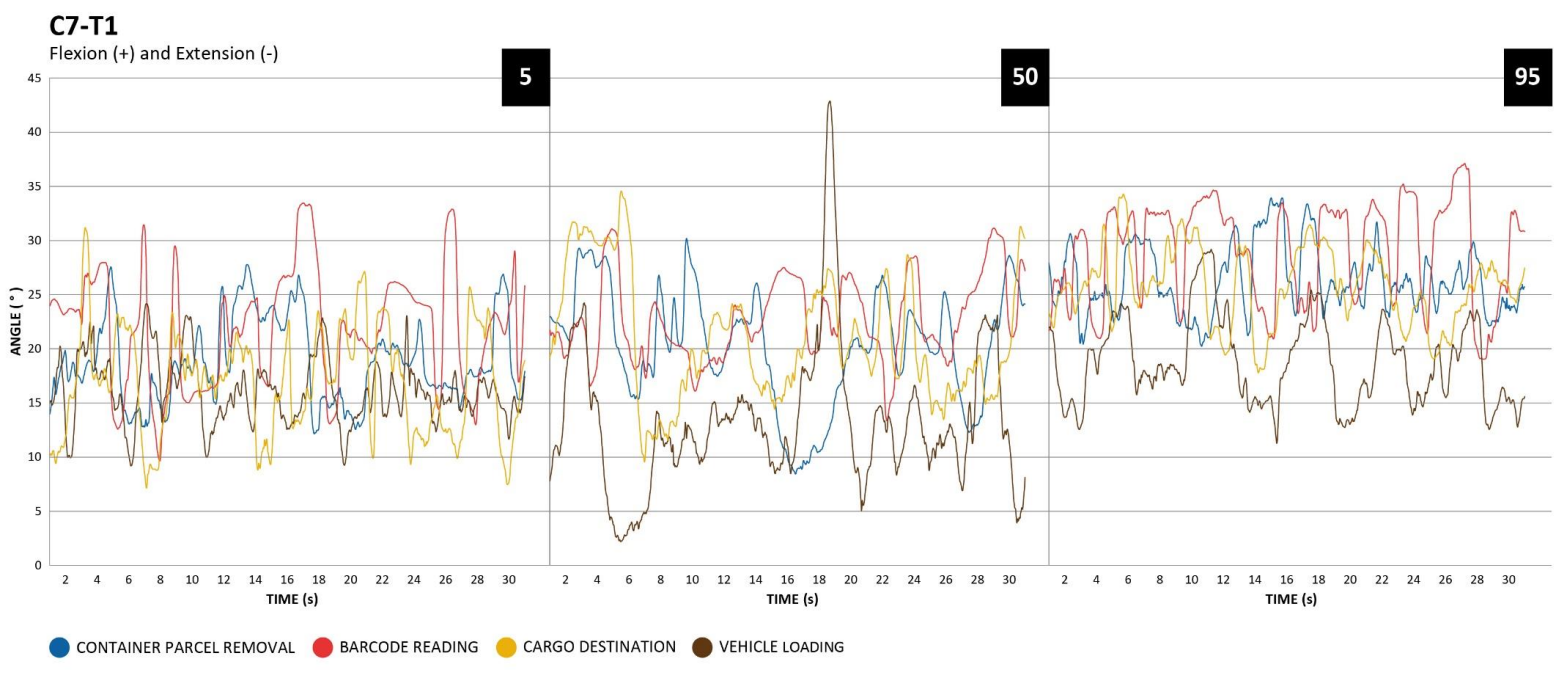
C7-T1, percentile 5, 50 and 95

**Figure 5:**
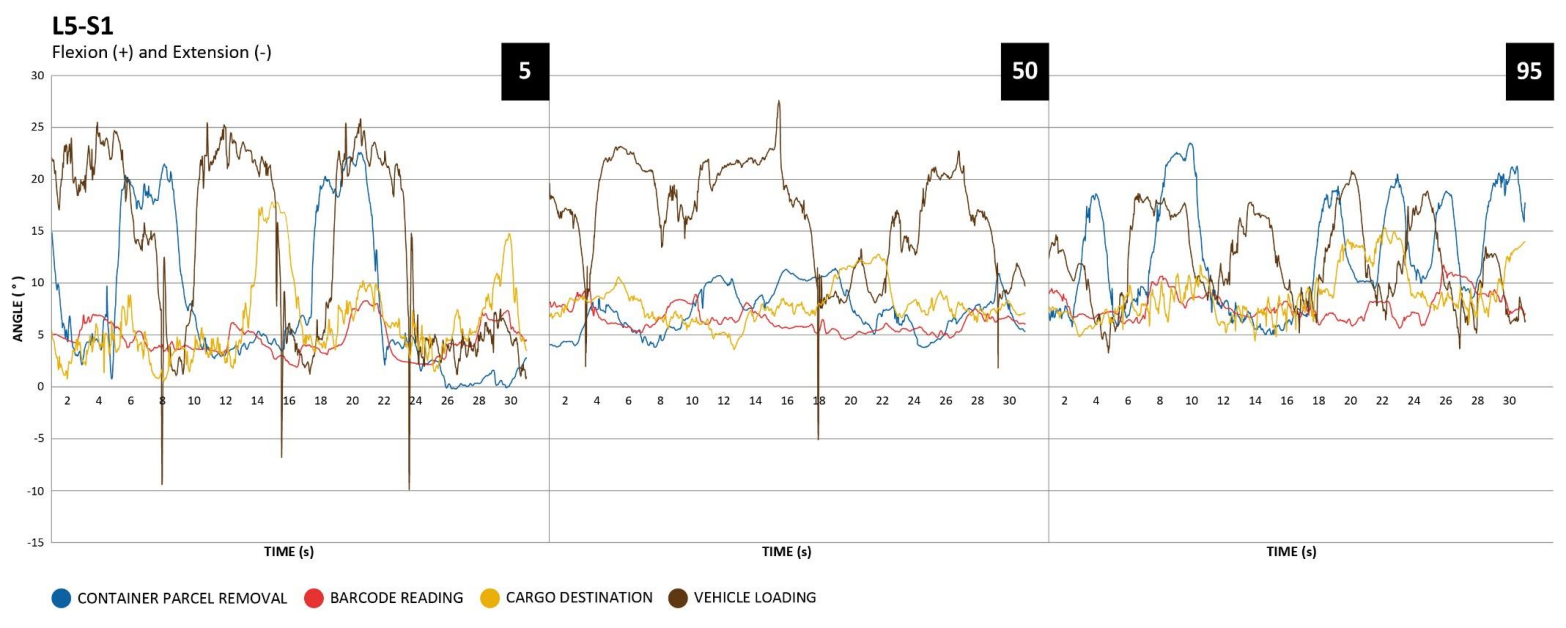
L5-S1, percentile 5, 50 and 95

The motion capture results can be observed in the following graphs (Figures 3-5(Fig. 3)(Fig. 4)(Fig. 5)) that represent each body part and respective percentile analyzed.

Regarding the movements of the right and left shoulder, flexion/extension can be observed in the graphs represented by Figure 3(Fig. 3).

In relation to the percentile 5, it can be observed higher shoulders anterior flexion angles in the activity of vehicle loading and container parcel removal. In addition, there was a predominance of use of the right upper limb.

The same can be observed in the 50 and 95 percentiles.

The percentile 50, although being left-handed, presented a right upper limb use pre-dominance in the vehicle loading activity and container parcel removal.

Even though there was a right upper limb use predominance, it was observed that in the bar code reading activity, the left upper limb obtained greater angular values due to its supportive function to explore the boxes surfaces to find the barcode.

#### C7-T1 segment

Regarding the C7-T1 segment, which reports the cervical anterior flexion, it is possible to observe the movements performed in the following graphs (Figure 4(Fig. 4)).

For the C7-T1 segment, barcode reading activity presented the highest angular values. In addition, the higher the percentile, the higher the angular values of cervical anterior flexion.

#### L5-S1 segment

The following graphs allow us to observe the movements of the L5-S1 segment, which reports the lumbar anterior flexion (Figure 5(Fig. 5)).

Regarding angular values related to the L5-S1 segment, vehicle loading and parcel removal from containers were the activities that obtained the highest values. It was also possible to observe repetitive behavior in the activity of vehicle loading.

Regarding the observed angular value averages and standard deviations (Table 2(Tab. 2)), it can be noted that in the C7-T1 segment there was an increment of these values as the height percentile increased. The same can be seen in the lower back in barcode reading and cargo destination activities. Concerning the shoulders, it presented higher angular values in the activity of barcode reading due to the support function on the left side to find the barcode on the package surface. 

## Discussion

According to Orr and Elyea (2006[25]), the ergonomic risk reduction process goes through two prerogatives, the identification of ergonomic risk factors and their elimination or reduction. Thus, this research aimed to identify the ergonomic risks of the order processing activity, through the use of qualitative and quantitative methods.

Among the qualitative methods, the Nordic musculoskeletal questionnaire was applied to 32 employees. The answers point to three regions that are mostly affected by MSDs: shoulders, lower back, and hands, respectively. Warnakulasuryia et al. (2012[35]), in their study with 250 postal workers, demonstrated that the regions most affected by musculoskeletal symptoms were lower back (24 %), shoulders (23 %), knees (18 %), neck (13 %), and wrists/hands (9 %). According to Hurley et al. (2012[15]), the most affected regions by MSDs in postal workers were shoulders and lower back. The main causes were related respectively to cargo lifting, variety of package shapes, and accidents due to climatic variations leading to falls on slippery floors.

Vehicle vibration was also mentioned by the author as a risk factor. According to Mattioli et al. (2011[22]), it may also be associated with hand illness in mail workers.

Regarding lower back pain, Radauceanu et al. (2019[28]) showed that lower back MSDs in parcel delivery workers were related to working time driving, cargo loading more than 3 kg/package, awkward postures, and increased demand for delivery work. The reports of pain in this region were also described by Sobti et al. (1997[30]), 39 % of the 3,920 retired male postal service workers presented low back pain positively related to high stature and loads lifting greater than 25 kg for more than 20 years.

Among the quantitative methods, the anthropometric evaluation showed that the evaluated group had a BMI average of 27.8 ± 3.7 kg/m^2^, being considered overweight in the WHO classification (WHO, 1998[36]). For postal workers, the increase in body mass was positively related to the illness of the knees and hips (Sobti et al, 1997[30]). Pain in these regions was also mentioned by the workers examined in our study, with the knees being the fourth region with the highest number of complaints. Anderson and Felson (1988[2]) demonstrated an important correlation between overweight, knee disease, and work with bent knee postures. Thus, it is necessary to reduce weight in order to promote the protective effect on knee cartilage and menisci (Gersing et al., 2017[12]).

This study also demonstrated that service time may have influenced more weight gain and loss of muscle mass than aging per se. This must be observed by the delivery company since overweight can increase the risk of musculoskeletal illness by 25 % to 68 % (Lin et al., 2013[19]; Gu et al., 2016[13]). Overweight can also be considered an important factor for occupational illness related to lifting loads (Colim et al., 2020[5]).

The movement analysis was performed by measuring shoulders' flexion and extension bilaterally, cervical anterior flexion movements, precisely in the C7-T1 segment, and anterior flexion movement of the lower back, in the L5-S1 segment. Regarding the shoulders, it can be observed that all observed percentiles exceeded the value of 60º of anterior flexion, especially in activities of container parcel removal and vehicle loading. The amount of time that shoulders remained at ≥60º of flexion may increase the MSDs risks, due to reduced blood flow to the interior of the muscles and its impact on the shoulder muscles and osteoligamentous structures (Pope et al., 2001[27]; Stenlund et al., 2002[31]; Leclerc et al., 2004[18]). In situations of anterior shoulder flexion between 30º and 90º, muscle activity can increase 84 % (Antony and Keir, 2010[3]).

In Brazil, according to the social security statistical yearbook, among the ICD (International Classification of Diseases) codes with the highest incidence in occupational diseases, the most incident ICD in 2017 were shoulder injuries (M75), followed by synovitis and tenosynovitis (M65) and lower back pain (M54) (AEPS, 2017[1]). In this scenario, it is noticed that the shoulder is one of the most affected joints when it comes to MSDs caused by work activities.

Cervical position, related to C7-T1 segment demonstrated a tendency of head anteriorization. The activity with the highest values for this tendency, comparing all percentiles, was barcode reading. It can be observed that the more the individual's height is, the greater is the head anteriorization in activities of barcode reading, cargo destination, and container parcel removal. The amount of time that the C7-T1 segment remained at ≥30º of anterior flexion may raise cervical MSDs risks (Iida and Guimarães, 2016[16]).

Regarding the L5-S1 segment, bending postures were observed, especially in container parcel removal and vehicle loading activities. In the analyzed employee group, the greater the height, the greater the angular values of anterior lumbar flexion in the activities of cargo destination and code bar reading. Working in bending postures is a risk factor for lower back pain (Miller and Fathallah, 2006[24]; DeBeeck and Hermans, 2000[6]; Lotters et al., 2003[20]). This risk was observed in England postal workers due to parcel lifting (Sobti et al., 1997[30]).

The MSDs risks pointed out in this study may lead the worker to affect negatively his physical health and generate increased absenteeism. Complaints of shoulder, cervical and lower back problems are common in this group (Mascarenhas and Barbosa-Branco, 2014[21]; Sobti et al., 1997[30]). This reality can be observed by the fact that workers in this sector belong to the group with the highest incidence of absenteeism in Brazil (Filgueiras, 2017[11]).

## Conclusions

Parcel delivery activity is becoming increasingly important in a scenario of progressive growth in e-commerce, and during the COVID-19 pandemic, this increase was highlighted. Companies in this sector value fast delivery and security of order integrity. However, for this to occur satisfactorily, there is a need for the worker responsible for handling and delivering parcels to be in good health. In this way, this study assessed musculoskeletal symptoms and movements of postal workers and identified risks of MSDs related to the activity of processing orders for delivery, proposing preventive measures.

The used methods were appropriate and complementary in understanding musculoskeletal symptoms and their relationship to the activity and anthropometry. The analyzed workers were overweight and presented musculoskeletal complaints in practically all body regions, with a greater prevalence in shoulders, hands, lower back, and knees.

Through motion capture, in real work situations, we could observe risks related to cervical protrusion, anterior trunk flexion, and shoulder flexion. In addition, repetitive lower back anterior flexion movements were identified, related to vehicle loading. The higher the stature, the higher was the increase in lumbar and cervical anterior flexion, risk factors that can lead these regions to illness.

Based on the results, some preventive measures were proposed: i) adjustment of the height of the container to reduce the anterior trunk flexion; ii) computer screen repositioning and adaptation of the bench´s height for barcode reading, in order to reduce the anterior cervical flexion; iii) adjustments of the vehicle interior to reduce the bending and stooped postures identified in the vehicle loading; iv) encourage the reduction of body mass through the practice of physical activities and improvement of the quality of the food consumed by postal workers in order to reduce MSDs (Gu et al., 2016[13]; Colim et al., 2020[5]).

Further studies should be conducted to find more ergonomic solutions to reduce the observed risks and evaluate this kind of activity performed in other locations and using different means of transport, such as bicycles and electric vehicles, focusing on the worker, but not disregarding the process. 

## Declaration

### Conflict of interest

The authors declare that they have no conflict of interest.

### Ethical approval

All procedures performed in studies involving human participants were in accordance with the ethical standards of the national research committee and with the 1964 Helsinki declaration and its later amendments or comparable ethical standards. The registration number of the Ethics Committee on Human Research is 4.655.606, CAAE: 40261320.2.0000.5322.

### Consent form

All participants involved in this study have signed an Informed Consent Form, according to items IV.3 of Resolution 466/12 of the National Health Council.

### Acknowledgments

The authors would like to thank the Brazilian Post and Telegraph Company (ECT), the National Council for Scientific and Technological Development (CNPq), ALGORITMI Research Centre (Portugal) and the Center for Design Management and Universal Design Laboratory of the Federal University of Santa Catarina (NGD-LDU/UFSC) for enabling this research.

This study was financed in part by the Coordenação de Aperfeiçoamento de Pessoal de Nível Superior - Brasil (CAPES) Finance [Code 001].

## Figures and Tables

**Table 1:**
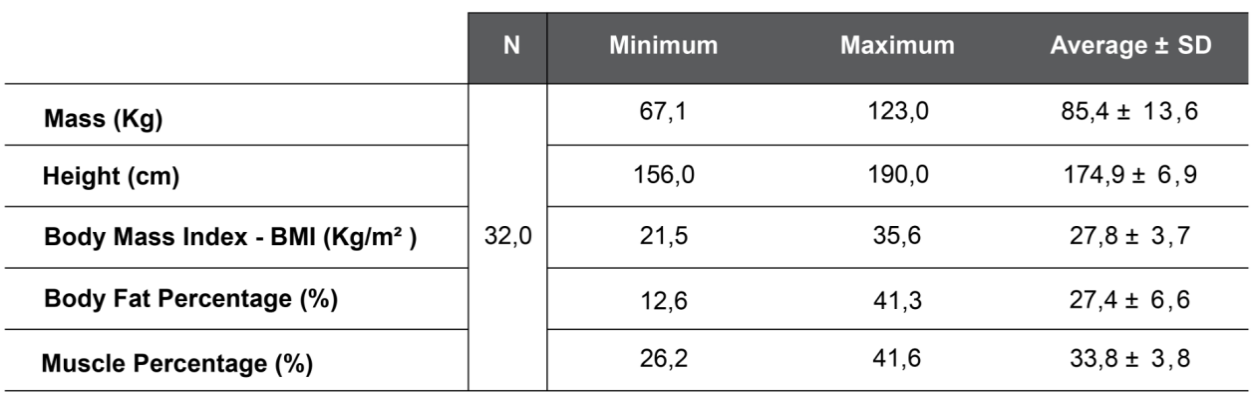
Anthropometric and BIA data

**Table 2:**
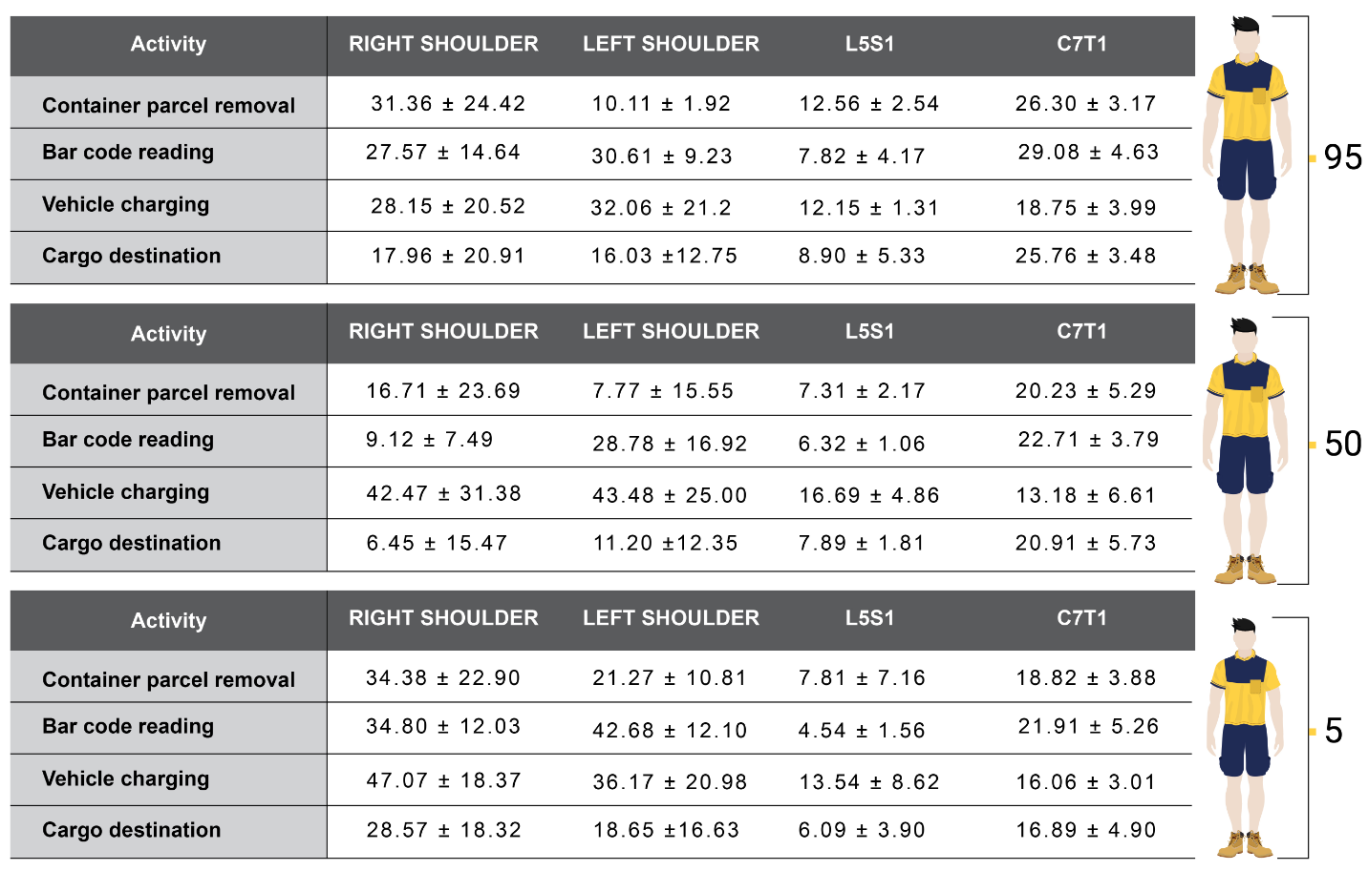
Average angles and respective standard deviation according to the activities performed: percentiles 95, 50 and 5
